# Morphofunctional evaluation of peripapillary retinoschisis associated with myopic posterior staphyloma and hyaloid traction: does it cause peripapillary vitreoretinal traction?

**DOI:** 10.5935/0004-2749.2021-0377

**Published:** 2022-09-06

**Authors:** Fillipe B. Borges, Leandro Cabral Zacharias, Sergio Luis Gianotti Pimentel, Leonardo Provetti Cunha, Mário Luis Ribeiro Monteiro, Rony Carlos Preti

**Affiliations:** 1 Division of Ophthalmology, Faculdade de Medicina, Universidade de São Paulo, São Paulo, SP, Brazil; 2 Department of Ophthalmology, Faculdade de Medicina, Universidade Federal de Juiz de Fora, Juiz de Fora, MG, Brazil

**Keywords:** Myopia, degenerative, Retinoschisis, Retinal detachment, Optical coherence tomography, Humans, Case reports, Miopia degenerativa, Retinosquise, Descolamento retiniano, Tomografia de coerência óptica, Humanos, Relatos de casos

## Abstract

This case report presents the details of a 33-year-old female patient who was referred to a specialized retina service because of mild vision loss in her right eye). The patient’s visual acuity was 20/25 in right eye and 20/50 in the left eye (; amblyopic); the spherical equivalent was -12.75 diopters (right eye) and -14.75 diopters (left eye). Multimodal retinal imaging showed peripapillary schisis in both the inner and outer retinal layers, grade II posterior vitreous detachment, and a tessellated fundus. Using Humphrey perimetry and MP-3 microperimetry, the functional evaluation indicated macular sensitivity within normal limits and decreased sensitivity in the peripapillary region, especially in right eye. The pattern-re versal visual evoked potential was measured. The N75 and P100 latency and amplitude in right eye were within normal values for checks of 1º. However, the amplitude was low for checks of 15′. Highly myopic patients who have posterior staphyloma that involves the optic nerve are susceptible to posterior hyaloid traction, and the resulting peripapillary vitreous traction may compromise vision.

## Abstract (pt)

Este relato de caso apresenta um paciente feminino de 33 anos encaminhado para um serviço especializado de retina devido à leve perda de visão em olho direito. A acuidade visual foi de 20/25 no olho direito e 20/50 no olho esquerdo, o equivalente esférico foi de -12,75 dioptrias e -14,75 dioptrias, respectivamente. Avaliações multimodais revelaram isquese peripapilar nas camadas internas e externas da retina, descolamento vítreo posterior grau II e fundo tesselado. Avaliação funcional com perimetria Humphrey e microperimetria MP-3 revelaram sensibilidade macular normais e diminuição da sensibilidade na região peripapilar, especialmente no olho direito. Potencial visual evocado de padrão reverso apresentou no olho direito latência e amplitude N75 e P100 dentro dos valores normais para verificação de 1º. Entretanto, a amplitude foi baixa para a de 15′. Pacientes alto míopes com esfiloma posterior envolvendo o nervo óptico são suscetíveis à tração da hialoide posterior. Portanto a tração vitreopapilar resultante pode causar comprometimento da visão.

## INTRODUCTION

Because of its vision-threatening complications, such as retinal detachment, macular hole, choroidal neovas-cularization, and myopic retinoschisis, the treatment of high myopia is particularly important^(1)^.

Myopic macular schisis was first described in 1999 by Takano and Kishi using optical coherence tomography (OCT) and later renamed *myopic tractional maculopathy*. The condition is characterized by a separation of the inner layers of the macula associated with posterior pole staphyloma but without blurred vision^(2)^. However, when macular retinoschisis is accompanied by serous retinal detachment, vision is compromised.

Peripapillary retinoschisis is a little-known subtype of maculoschisis that is observed in patients with high myopia. Carbonelli et al. reported the first case without the assistance of multimodal imaging^(3)^.

In this study, we used multimodal imaging to conduct a morphofunctional analysis of high myopia with posterior pole staphyloma associated with peripapillary retinoschisis that was likely caused by a combination of staphyloma and hyaloid traction and apparently resulted in peripapillary vitreoretinal traction (PVT).

## CASE REPORT

A 33-year-old female patient was referred to a specialized retina service due to mild vision loss in her rigth eye (OD). The patient’s visual acuity was 20/25 in OD and 20/50 in the left eye (OS; amblyopic); the spherical equivalent was -12.75 diopters (OD) and -14.75 diopters (OS).

We measured central corneal thickness (OD 530 µm; OS 527 µm) using an ultrasonic contact pachymeter (OcuScan RxP Ophthalmic Ultrasound System, Alcon Laboratories). An optical biometer (IOL master 500, Carl Zeiss Meditec AG, Jena, Germany) was used to measure axial length (OD 28.4 mm; OS 29.88 mm)

**Figure 1:**
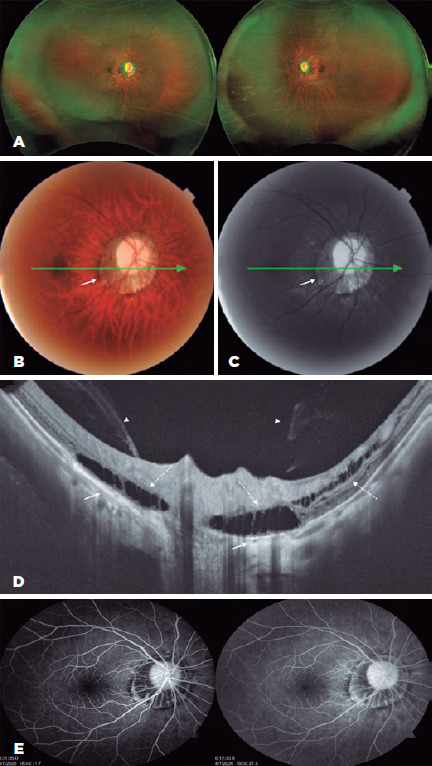
(A) Ultra-wide-feld (UWF) retinography shows peripapillary posterior staphyloma with peripheral microcytic degeneration. (B) Retinography of 45° indicates mild disc pallor; mild difuse rarefaction of the retinal pigment epithelium (RPE), with great evidence of the choroidal vessels; and 360° peripapillary atrophy (small continuous arrow). (C) Red-free image shows a hyperreflective area indicating atrophy of the peripapillary RPE (small continuous arrow). (D) SS-OCT reveals maintenance of vitreous attachment to the optic nerve (arrowhead), with cleavage of the peripapillary outer retinal layers characteristic of peripapillary retinoschisis (dotted arrow) and RPE atrophy (continuous arrow). (E) Fluorescein angiography shows the peripapillary window defect.

On ultra-wide-field retinography (Daytona, Optos, Dunfermline, UK), both eyes demonstrated peripheral microcytic degeneration associated with posterior staphyloma, increased fundus tessellation, and peripapillary atrophy (Figure 1A). This was confirmed on 45° retinography and red-free imaging (Triton® swept-source OCT [SS-OCT], Topcon, Tokyo, Japan; Figure 1B,C). Peripapillary schisis in both the inner and outer retinal layers and grade II posterior vitreous detachment (PVD) were observed on SS-OCT (Triton®, Topcon), and the macula presented a tessellated fundus (Figure 1D). Fluorescein angiography did not reveal any signs of swelling in the macula or optic disc (Visucam, Carl Zeiss Meditec, Jena, Germany; Figure 1E).

**Figure 2:**
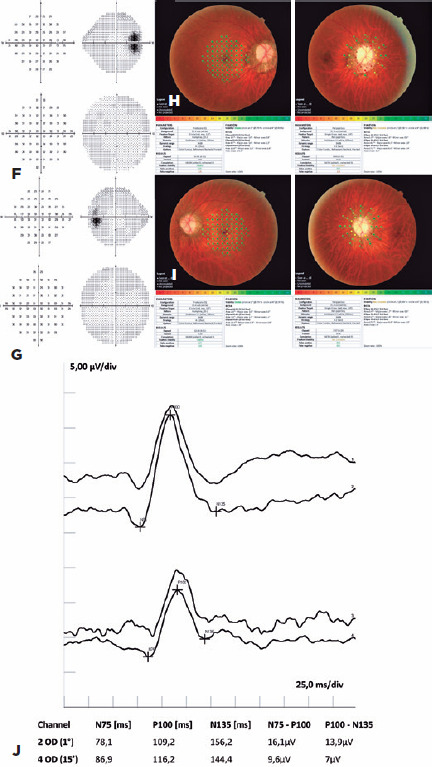
(F, G) Blind spot enlargement is shown on 24-2 and 10-2 automated perimetry of OD and OS. (H, I) Microperimetry indicates normal and decreased macular and peripapillary sensitivity, respectively. (J) Reversal visual evoked potential of OD shows N75 and P100 latency and an amplitude within normal values for checks of 1°, as well as normal latency but low amplitude for checks of 15′.

Using both the 24-2 and the 10-2 strategy, the functional evaluation conducted with Humphrey perimetry (Carl Zeiss Meditec, Dublin, CA, USA) showed macular sensitivity within normal limits and decreased sensitivity around the optic disc (Figure 2F,G). Macular sensitivity was also within normal limits on microperimetry (MP-3, cavitation, tractional detachment of the internal limiting membrane, macular holes, retinal detachment, and neovascular choroidal membranes. However retinoschisis (dehiscence of the retinal layers) is an unusual and intriguing finding^(4)^. As shown by Ohno-Matsui, in patients with myopia, PVD progresses asymmetrically and is associated with scleral curvature (different points of traction). This explains manifestations such as retinoschisis^(5)^, which can compromise vision when severe^(6)^.

NIDEK Co. Ltd., Aichi, Japan); however, sensitivity was decreased in the peripapillary region of both eyes, especially in OD (Figure 2H,I). A RETI-scan device (Roland Consult, Wiesbaden, Germany) was used to measure the pattern-reversal visual evoked potential (PR-VEP) following the guidelines of the International Society for Clinical Electrophysiology of Vision. The N75 and P100 latency and amplitude in OD were within normal values for checks of 1º. However, the amplitude was low for checks of 15′ (Figure 2J).

## DISCUSSION

In patients with high myopia, findings expected on OCT include paravascular internal retinal cleavage, lamellar cysts and holes, intrachoroidal peripapillary

Retinal peripapillary traction occurred in our patient only in the optic nerve region because of the localized nature of type III peripapillary staphyloma (Curtin’s classification)^(7)^. Figure 1D shows that this type of staphyloma seems to predispose to stage II of PVD, where the sclera is the most outpouched posteriorly, producing the appearance of anteroposterior PVT (horse rein effect) and leading to retinoschisis of almost 360°^(5,8)^.

Interestingly, even in the absence of myopic macular schisis, our patient displayed mild visual loss. The lack of changes in macular sensitivity on the microperimetry and automated perimetry to explain this loss in visual acuity raised the hypothesis of PVT, a condition that is relatively recently described and poorly understood. Conceivably, severe PVT could lead to vision loss via peripapillary schisis^(6)^.

We attempoted to validate our hypothesis by submitting the patient to PR-VEP testing. Although the response was normal for checks of 1°, the amplitude was low for checks of 15′. Clinical PR-VEP testing should be interpreted with consideration of the activity of the retinal visual field; thus, a check size of 1° elicits a mostly parafoveal response, whereas a check size of 15′ elicits a foveal response. In their work, Soares and coworkers reported that patients with mild vision loss may occasionally have normal PR-VEP findings, indicating integrity of the visual pathway^(9)^. Thus, in this case, the peripapillary findings observed on SS-OCT associated with functional tests led us to believe that PVT is present.

In patients with moderate and high retinal myopic degeneration, scotopic and photopic electroretinographic (ERG) and multifocal ERG responses are reduced and delayed, and the number of macular cones is reduced. Unfortunately, our patient declined to undergo multifocal ERG.

In line with this hypothesis, Cunha et al. recently reported a case of an 85-year-old man with a progressive visual loss secondary to severe vitreopapillary traction. In their case, the SS-OCT B-scans revealed circumpapillary anteroposterior dense vitreous traction strands on the optic disc leading to peripapillary retinoschisis, which affected both inner and outer retinal layers. Posterior vitrectomy was performed, and the patient achieved great visual improvement (20/60 to 20/25). The authors concluded that, as in our case, we found that the mechanical deformation onto the optic disc and peripapillary retina could impair the the axoplasmic flow and neuroretinal sign transmission, resulting in visual loss^(10)^.

Finally, the blind spot in OD in our patient was increased on perimetry (Figure 2F), whereas the sensitivity of the peripapillary retina was reduced on microperimetry (Figure 2H). We believe that the observed loss in peripapillary function was due not only to retinoschisis but also to external retinal atrophy in the same location, as suggested by the window defect revealed on fluorescein angiography (Figure 2E).

Type III posterior pole staphyloma involving the optic nerve may present with posterior hyaloid traction, resulting in extensive peripapillary schisis. The resulting peripapillary vitreous traction may compromise vision.
